# Compound‐specific stable hydrogen isotope (*δ* ^2^H) analyses of fatty acids: A new method and perspectives for trophic and movement ecology

**DOI:** 10.1002/rcm.9135

**Published:** 2021-06-25

**Authors:** Matthias Pilecky, Katharina Winter, Leonard I. Wassenaar, Martin J. Kainz

**Affiliations:** ^1^ WasserCluster Lunz ‐ Biologische Station Dr. Carl‐Kupelwieser Promenade 5 Lunz/See 3293 Austria; ^2^ Department of BioMedical Research Danube University Krems Krems 3500 Austria; ^3^ International Atomic Energy Agency Vienna International Centre Vienna 1400 Austria

## Abstract

**Rationale:**

Compound‐specific stable isotope analysis (CSIA) is a powerful tool for a better understanding of trophic transfer of dietary molecules in and across ecosystems. Hydrogen isotope values (*δ* ^2^H) in consumer tissues have potential to more clearly distinguish dietary sources than ^13^C or ^15^N values within and among habitats, but have not been used at the fatty acid level for ecological purposes.

**Methods:**

Here we demonstrate a new online high‐capacity gas chromatography–isotope ratio mass spectrometry technique (^2^H‐CSIA) that offers accurate and reproducible determination of *δ* ^2^H values for a range of fatty acids from organisms of aquatic food webs.

**Results:**

We show that lipid extracts obtained from aquatic sources, such as biofilms, leaves, invertebrates, or fish muscle tissue, have distinctive *δ* ^2^H values that can be used to assess sources and trophic interactions, as well as dietary allocation and origin of fatty acids within consumer tissue.

**Conclusions:**

The new ^2^H‐CSIA method can be applied to evaluate sources and trophic dynamics of fatty acids in organisms ranging from food web ecology to migratory connectivity.

## 1 INTRODUCTION

Tracing trophic transfer of dietary energy sources and molecules in and among aquatic and terrestrial food webs is critical for understanding and quantifying trophic interactions of species. Stable isotope analyses (e.g. *δ* ^13^C, *δ* ^15^N, *δ* ^34^S) of primary producers and consumer tissues (e.g. algae, leaves, feathers, muscle tissue, etc.) have been used since the 1980s to construct trophic models for terrestrial, freshwater, and marine ecosystems.^1^, ^2^, ^3^, ^4^, ^5^ Bulk tissue *δ* ^2^H or bulk lipid *δ* ^2^H analyses have been applied to track animal migration patterns and provenance of lipid synthesis^6^, ^7^, ^8^ and offer low‐cost routine approaches, but suffer from several intrinsic challenges: (i) bulk tissue isotopic averaging of all molecular components, such as carbohydrates, proteins, and lipids, all of which undergoing anabolic and catabolic processes at different turnover times, thus distorting the bulk *δ* value depending on the current physiological state of the biological sample; (ii) isotopic fractionation by biochemical processes; (iii) bulk stable isotopic overlap of available food sources; and (iv) specific to any H isotope exchange with environmental water.^9^, ^10^, ^11^ In the past decade, technological advances in compound‐specific isotope analysis (CSIA) of specific molecules extracted from tissues (e.g. essential or nonessential amino acids) have offered a deeper view of trophic transfer, and trophic ecology in general, but these discrete molecular (or compound‐specific) stable isotope assays require specialized preparation and complex gas or liquid chromatographic interfaces to combustion‐based isotope ratio instruments, and, so far, are largely limited to ^13^C or ^15^N.^12^, ^13^ More recently, the use of hydrogen isotopes (*δ* ^2^H) has provided a promising approach owing to large and predictable H isotope patterns between aquatic and terrestrial sources, season, i.e. differences in precipitation amounts and ambient temperature, as well as the significant bulk tissue H isotopic differences commonly observed between terrestrial and aquatic primary producers.^10^, ^14^, ^15^ The extension of H isotopes towards compound‐specific assays of fatty acids has long been seen as encouraging, as has been shown for amino acids.^16^, ^17^ Furthermore, a recent study comparing bulk plasma fatty acid composition with feather bulk *δ* ^2^H could infer relative inputs to swallows from terrestrial and aquatic insects.^18^ Still, the use of ^2^H‐CSIA for lipids in trophic ecology is largely unexplored and mainly focused on plant lipid and wax extract studies,^17^, ^19^, ^20^, ^21^ or paleobiology and geosciences.^22^, ^23^

Fatty acids are quantifiable tracers used to assess the trophic transfer of specific dietary energy sources, such as bacterial‐, algal‐, or terrestrial‐derived fatty acids.^24^, ^25^, ^26^ However, it is problematic to differentiate amongst these different dietary sources of fatty acids; for example, the essential polyunsaturated fatty acids (PUFA) α‐linolenic (ALA) and linoleic (LIN) acids are synthesized in terrestrial and also aquatic primary producers,^27^ and hence cannot be used as clear dietary source‐specific markers. The application of *δ* ^2^H is exceptionally promising compared to *δ* ^13^C because large bulk H isotopic differences have already been seen between individual diet sources and consumers both spatially and temporally.^8^, ^28^, ^29^, ^30^ In most lipids, the C‐H bonds of the alkyl chains are chemically stable and nonexchangeable with ambient water, hence preserving the original H isotope source signal, but may occur over much longer geological timescales,^31^ in contrast to the rapid H isotope exchange with water, for example, in carbohydrates. Thus, *δ* ^2^H values of nonessential components will reflect (with biological H isotope fractionation) the deuterium abundance of local environmental water, while *δ* ^2^H values in essential components should resemble those of the consumer's diet, as has been shown for amino acids.^16^ In general, lipid synthesis leads to significant ^2^H depletion (on average 150 ‰) in the synthesized organic matter, yet environmental water and lipid *δ* ^2^H values are usually strongly correlated.^20^, ^32^ This ^2^H depletion can be partially explained by H isotopic fractionation due to recurrent elongation and saturation,^19^ but is also strongly dependent on the physiological processes of the individual species. When comparing *δ* ^2^H values of fatty acids between diets and consumers, four key processes with high potential for H isotope fractionation have to be considered: (1) H isotope fractionation during dietary fatty acid uptake and allocation to individual tissues; (2) the H isotope composition of cellular water, acetate, and proton carrying cofactors such as nicotinamide adenine dinucleotide phosphate, which are used for fatty acid synthesis and modification; (3) H isotope fractionation by enzymes involved in the lipid biosynthetic pathways themselves, which in general utilize lighter substrates faster than their heavier isotopologues; and (4) isotope fractionation due to fatty acid transport and metabolism.^33^ As a result, fatty acids that are synthesized by a consumer are often ^2^H‐depleted compared to diet, while fatty acids that are not needed by a consumer and thus catabolized become comparatively enriched in ^2^H.

Utilization of *δ* ^2^H values of fatty acids thus requires thorough knowledge of species physiology to reveal possible H isotope fractionation pathways along trophic transfer and within or amongst individuals.^34^ For example, the enzymatic processes for conversion of precursors to long‐chain PUFA involve elongation and desaturation, and possibly to some extent retro‐conversion back to short‐chain PUFA, which seems likely to be a process that alters the *δ* ^2^H values inessential fatty acids. In addition, source water and the seasonal variations in ^2^H content of water sources within the sampling area need to be considered to establish baseline H isotope conditions. A major technological and analytical challenge in achieving *δ* ^2^H assays of individual lipids in environmental samples is the exceedingly low natural abundance of ^2^H in combination with low contents of the lipids of interest for most samples. This requires improved detection limits and the need for highly specialized chemical and isotopic analyses that inherently have larger uncertainties than are commonly expected for routine bulk sample deuterium measurements (e.g. less than ±2–4 ‰) due to greater analytical complexity.

Previous attempts to use *δ* ^2^H in an ecological context failed mainly due to the low H_2_ signal intensity of the mass spectrometer, which led to unreliable or highly uncertain results that were difficult or ambiguous to interpret. The aim of the study reported here was twofold. First, we present a new analytical method and optimized isotope ratio mass spectrometry (IRMS) workflow that utilizes high‐capacity gas chromatography (HC‐GC) to isolate essential and nonessential lipids from environmental samples, along with online thermochemical conversion to H_2_ gas, and introduce the setup and provide the parameters for reliable and reproducible determinations of *δ* ^2^H values by IRMS. Second, we provide examples of ^2^H‐CSIA for aquatic food web ecology to better understand how dietary fatty acid sources are trophically transferred and subsequently retained in consumers.

## 2 MATERIALS AND METHODS

### 2.1 Environmental samples

A suite of natural lipid samples extracted from leaves, stream periphyton, macroinvertebrates, and fish that were previously sampled, preserved, and stored^35^ was used to optimize the analysis and evaluate the potential of compound‐specific *δ* ^2^H analysis of fatty acids. Briefly, the environmental samples were collected from the subalpine Ybbs River catchment near Lunz am See, Austria (47°45′ N, 15°12′ E), between July and October 2016, were subsequently freeze‐dried and stored (Virtis Genesis freeze dryer). All samples were weighed and homogenized using a glass rod or food processor (leaves). Lipids were extracted from the samples as described elsewhere,^36^ using a chloroform–methanol mix (2:1 v/v) and sonication. Samples were vortexed and centrifuged three times to remove nonlipid phases. The extracted lipids were evaporated to a final volume (1.5 mL) using a stream of N_2_ gas. For fatty acid methyl ester (FAME) formation suitable for GC, the samples were incubated with a sulfuric acid–methanol mix (1:100 v/v) for 16 h at 50°C followed by the addition of an equal normality of KHCO_3_ and hexane. Samples were manually shaken, vortexed, and then centrifuged. The supernatant organic FAME layers were collected, pooled, and again concentrated under N_2_ gas to 30 μL to be used for GC characterization and H isotope analysis. Samples were stored under inert N_2_ atmosphere at −80°C.

### 2.2 FAME analysis using GC

Fatty acid samples were first characterized using a Thermo Trace 1310GC (Thermo Scientific, Waltham, MA) equipped with a liquid autosampler. FAME were GC‐resolved using a VF‐WAXms 60 m column, 0.25 mm inner diameter (ID), 0.25 μm film thickness (FT) (CP9207, Lot NLR0669603; Agilent Technologies, Santa Clara, CA). For H isotope analyses, a high‐capacity VF‐WAXms 30 m column, 0.32 mm ID, 1 μm FT (CP9211, Lot NLT0709896; Agilent Technologies) was used. For both columns, the GC injector port was held at 250°C. In spitless mode, 3 μL of each sample was injected which was previously determined to be the maximal volume without backflash using a split/splitless liner with single taper (4 mm × 6.3 mm × 78.5 mm, 453A1355, Thermo Scientific) following activation of the purge flow after 1 min. For the thinner 60 m column, the GC temperature program started at 80°C for 2 min and ramped up by 30°C min^−1^to 175°C, then by 5°C min^−1^to 200°C, and finally by 2.4°C min^−1^ to 250°C which was maintained for 30 min for a total GC run time of 62 min. The temperature program for the shorter high‐capacity 30 m GC column used for isotope analyses started at 80°C for 2 min, was ramped up by 30°C min^−1^ to 175°C, and then by 5°C min^−1^ to the 240°C maximal temperature, and held for 35 min. The total run time for the 30 m HC‐GC column used for H isotope analysis was 52 min.

### 2.3 *δ* ^2^H measurements

For the *δ* ^2^H isotope analyses, the GC‐resolved FAME were passed through a high‐temperature (1400°C) thermochemical carbon‐conditioned open ceramic reduction reactor (hydrothermal carbonization; HTC) which quantitatively pyrolyzed the FAME molecules to pure H_2_ gas, with the sample carbon reduced and retained as graphite in the reactor. The HTC reactor was preconditioned using 2 μL hexane injections before each GC sample sequence of 40–60 samples, followed by reverse flushing with He for 1 h. Each sample batch session was preceded with triplicates of USGS70 and USGS71 standards for data normalization purposes and performance testing. After every 10 unknown sample runs, these high and low *δ* ^2^H standards were repeated to ensure system stability and to check for instrumental drift.

The He flow rate through the HTC reactor was kept constant at 1.1 mL min^−1^ to ensure stable and quantitative conversions to H_2_. Ionization energy of the IRMS source was optimized to obtain the highest total H_2_^+^ ion current yield without excessive formation of He^2+^ ions. An H_3_^+^‐factor determination was performed before and after each measurement batch sequence using a dilution series of reference gas and was found to be low and stable (3.0 ± 0.1 ppm/V). FAME samples were identified on the IRMS H_2_ chromatograms by a comparison of their retention times with those of known standards (37‐component FAME mix, 47885‐U, Supelco; Sigma‐Aldrich, Bellefonte, PA). Peak amplitudes refer to the total ion intensity of mass 2 (^1^H_2_). Since the 30 m column was found more suitable for the intended purpose, all environmental samples for ^2^H were analyzed using the 30 m HC‐GC setup.

The *δ* ^2^H values of reagent methanol used in sample preparation were determined by cleaning the autosampler needle with toluene, followed by dipping the needle into four different aliquots from the bottles used for methylation without any aspiration and immediate injection into the GC equipped with a VF‐1701ms (30 m, 0.32 mm ID, 1 μm FT, Agilent Technologies). The GC inlet was heated to 250°C and this injection was performed in split mode using a ratio of 1:5. The starting temperature of 40°C was held for 1 min followed by an increase to 150°C at a rate of 30°C min^−1^. Each vial was measured eight times and *δ* ^2^H values of methanol were found to be consistent at −146.8 ‰ ± 2.6 (VSMOW). Other methods such as injection of different concentrations of methanol in toluene or variation of split ratio provided nearly identical average *δ* ^2^H values, but with significantly higher standard deviations (data not shown). Thus, it can be assumed that no H isotopic fraction for methanol was induced. It was reported that the effect of H isotopic fractionation induced by the process of fatty acid methylation is usually below the detection limit of IRMS systems.^37^ For the introduced methyl group, we performed “methanol corrections” to avoid biases due to potentially large isotopic differences between the *δ* ^2^H value of the methanol and those of fatty acids in the sample:

(1) $$\left(H_{n}+2\right)\;\delta^{2}H_{\text{FAME}}=\left(H_{n}-1\right)\;\delta^{2}H_{\mathrm{FA}}+3\;\delta^{2}H_{\mathrm{Me}}$$

where H_*n*_ is the number of H atoms for each fatty acid, *δ* ^2^H_FAME_ the observed value for the FAME, *δ* ^2^H_FA_ the desired value for the target fatty acid and *δ* ^2^H_Me_ the previously determined value of the methanol used for transmethylation. All values reported here refer to the *δ* ^2^H_FA_ values.

### 2.4 Data analysis

The *δ* ^2^H values of individual FAME compounds were determined using automated peak integration by defining 1 mV/s as the start and 0.5 mV/s as end point of the sample H_2_ peak. Background was automatically calculated by dynamic background calculation using a step width of 150 s. In the tests described below, individual background calculations with varying history times, dynamic background calculation with larger or smaller step widths, or BaseFit algorithm with a step width of 200 s and different smoothing factors were also tested.^38^ In our FAME samples, peaks were only considered acceptable for isotope data analysis if the H_2_ amplitude exceeded 450 mV and the peak area was more than 4 V s. All H_2_ peaks were validated manually and start‐and‐end‐point corrected, as necessary. While manual background correction can be effective, it is subject to individual operator preferences and requires experience. For this study, no manual background corrections were performed. The HC‐GC setup used in this study moreover did not allow for a clear separation between C18:1n‐7 and C18:1n‐9, and therefore both peaks were combined as one area thereby providing a general weighted‐average *δ* ^2^H value for C18:1 isomers.^38^

### 2.5 Reference materials and normalization

All H isotope analyses were calibrated against the FAME‐C20 standards USGS70 and USGS71 having assigned *δ* ^2^H (VSMOW) values of −183.9 ± 1.4 ‰ and −4.9 ± 1 ‰, respectively (https://isotopes.usgs.gov/lab/referencematerials/USGS70-USGS71-USGS72.pdf) and 2‐point normalization. Both USGS reference materials were shipped as freeze‐dried substances and stored sealed under inert N_2_ atmosphere at −20°C, as recommended (Arndt Schimmelmann, University of Indiana). Before each measurement series, the USGS standards were prepared freshly, dissolved to a final concentration of 500 mg/L in hexane and used for VSMOW‐SLAP normalization. The USGS reference materials were each run three times at the beginning and the end of each run. No instrumental H isotopic drift was observed in the USGS standards over the course of sample batches. Linearity corrections for H_3_^+^‐factor were determined and applied to all sample peaks by using automated CONFLOW H_2_ reference injections as part of the IsoDat instrument package. All *δ* ^2^H values are reported in the usual *δ* notation relative to Vienna Standard Mean Ocean Water:

(2) δH2FA=H2/H1SampleH2/H1VSMOW−1×1000

### 2.6 Statistical analysis

All data were processed using the R packages rstatix, ggplot2, ggpubr, and survMisc (R Project, Version 3.6.2). Results are presented as fully error propagated mean ± standard deviation. Error propagation included the δ^2^H uncertainty in VSMOW2 (±0.3 ‰), USGS 70/71 (±1.4 ‰/±1.0 ‰), and the standard deviation of replicated control samples, as applicable. Multiple group comparison of the compound‐specific *δ* ^2^H value for each specimen type was performed using ANOVA and Tukey's HSD *post‐hoc* test. Principal components analysis (PCA) was performed to examine different isotopic patterns among allochthonous and autochthonous diet.

## 3 RESULTS

Analytical performance of IRMS for *δ* ^2^H quantification was determined by two independent series of four consecutive measurements for each USGS standard. The typical standard deviation for USGS70 was ±2.7 ‰, and for USGS71 was ±1.3 ‰, resulting in a propagated *δ* ^2^H uncertainty of ±3.2 ‰ and ±2.2 ‰, respectively.

**FIGURE 1:**
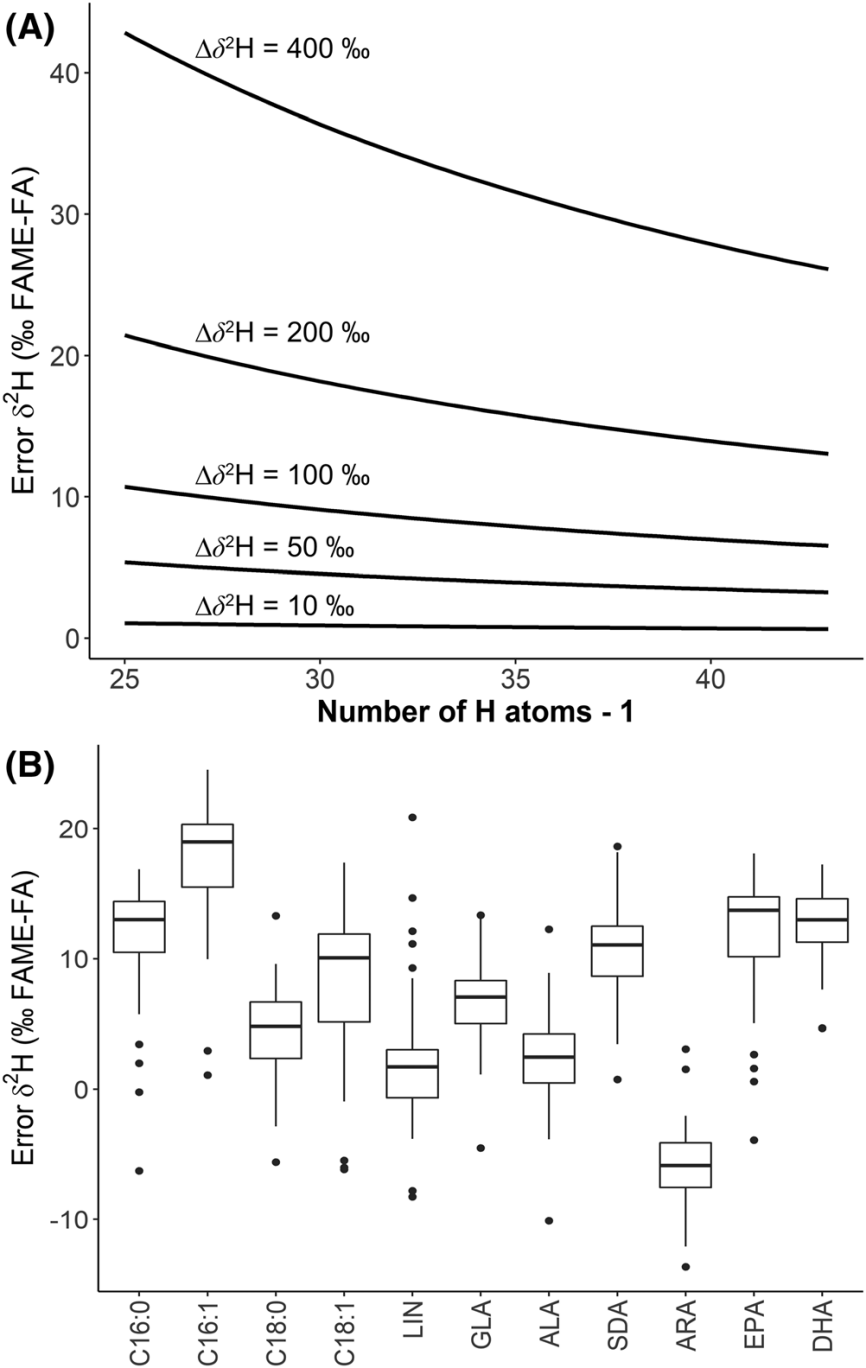
Assessment of fatty acid *δ* ^2^H bias from preparative methylation. (A) Theoretical shifts between the derivatized FAME and original fatty acid of interest as a function of number of H atoms of the acyl chain as dependent on the isotope difference between methanol used for derivatization and the fatty acid in the sample. (B) Observed differences between FAME and fatty acid after correction for methyl group for data. Significant biases in *δ* ^2^H, as for LIN and ARA or ALA and EPA/DHA, may lead to data misinterpretation and reduce comparability between different laboratories

The impact of methylation was compared to theoretical mass balance calculations of H isotope shifts between FAME and fatty acid as a function of the number of H atoms of the acyl chain depending on the difference from the *δ* ^2^H values of the methanol used (Figure 1A). We observed that the methyl group significantly shifted the observed *δ* ^2^H value of FAME (Figure 1B). The observed differences between the laboratory‐derivatized FAME and original fatty acid of interest were eliminated after a correction for the methyl group. Uncorrected differences in *δ* ^2^H, such as for LIN and arachidonic acid (ARA) or ALA and eicosapentaenoic acid (EPA)/docosahexaenoic acid (DHA), might lead to misinterpretation and reduce the comparability between different laboratories and even batches of methanol used for analysis; hence we recommend that a methyl bias correction is routinely applied.

**FIGURE 2:**
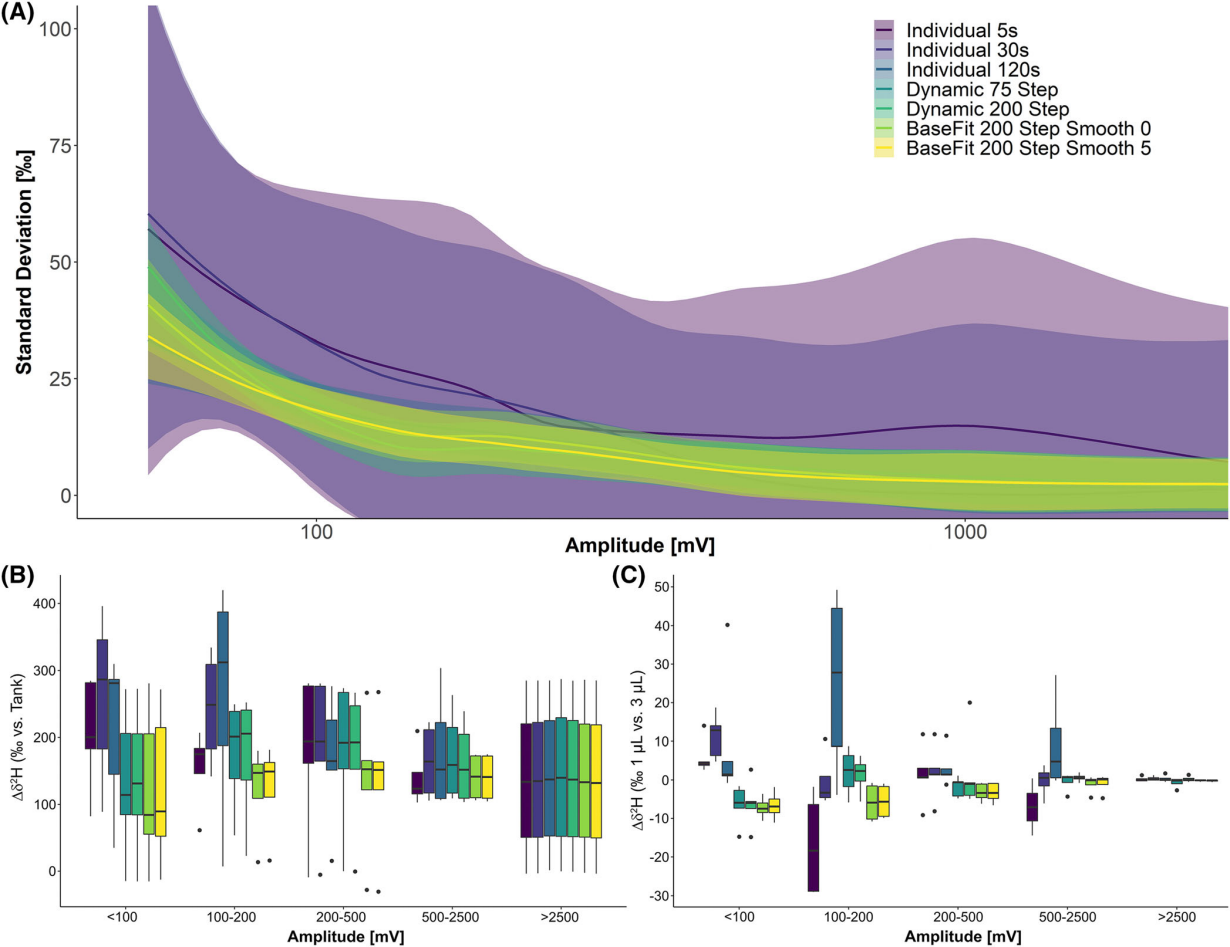
Impact of background correction on *δ* ^2^H values. (A) Individual background calculation resulted in peaks with high standard deviation regardless of peak intensity, while dynamic and BaseFit algorithm produced acceptable results if peak amplitude exceeded 1000 mV, with increase in standard deviation to about 10 ‰ at 250 mV and large increase below. (B) Background correction influences precision and the *δ* value of individual peaks. While background correction showed little effect, >2500 mV biases increased with reduced signal intensities. (C) Influence of varying signal intensities due to varying injection volumes on *δ* ^2^H values. BaseFit appeared to be most robust but required tuning for each setup and sample matrix. For peaks >500 mV of amplitude, dynamic background calculation performed equally well

To ensure robust H isotope analyses for more complex mixtures of FAME, a fish liver sample was measured four times sequentially using 1 μL and once using 3 μL to check for H isotope stability and sample concentration dependence. H_2_ peaks (total ion current) were selected between 700 and 2000 s, the first chosen H_2_ peak being identified as C14:0‐Me and the last one as EPA‐Me. For this evaluation only baseline‐separated peaks were selected, and all incompletely separated peaks such as those of the C18:1 isomers were excluded. In total, 24 peaks were identified and used for evaluation and classified into five groups according to their IRMS signal amplitude: <100 mV: 6 peaks; 100–200 mV: 4 peaks; 200–500 mV: 6 peaks; 500–2,500 mV: 4 peaks; >2,500 mV: 4 peaks. These peaks were automatically selected, and H_2_ backgrounds automatically calculated using either individual background mode which uses the lowest background values found in the chosen history time, dynamic, or the BaseFit algorithm which adjusts the background over the entire ^2^H chromatogram. Detailed explanations of peak selection and background calculation algorithms can be found elsewhere.^38^ No significant differences between the background calculation modes were observed regarding peak standard deviation (±2.2 ‰) or uncorrected *δ* ^2^H values for peaks with an amplitude of more than 2500 mV. Below this threshold, background calculations had a significant effect on both the standard deviation and uncorrected *δ* ^2^H values, despite the applied automated linearity correction. Individual peak background calculation is prone to variation within the background and resulted in multiple outliers that significantly increased standard deviation and severely altered the ^2^H/H isotopic ratio. The dynamic and BaseFit algorithms performed equally well for peaks with an amplitude >500 mV with a maximum average standard deviation of ±4.8 ‰. Unsurprisingly, a further decreasing sample peak amplitude was inversely correlated to a rise in standard deviation that exceeded 30‰ for some peaks <100 mV (Figure 2A). These results were consistent with poorer performance for low‐level peaks close to background noise, but also possibly with flow variations in the system.

Large variations of sample *δ* ^2^H values based on the background calculation algorithm used were observed for peaks below 500 mV amplitude, with unacceptable deviations of >100 ‰ for some small peaks (Figure 2B). Individual background calculation was especially prone to variations in the H_2_ background due to the different volumes of injected sample (Figure 2C). These data clearly show that peaks above 1 V amplitude provided the most reliable results, but requiring sufficient sample material. Furthermore, H_2_ background calculation algorithms must be carefully tested for each IRMS setup.

### 3.1 Comparison of GC column performance for GC/^2^H‐IRMS

To evaluate the influence of GC column type on GC/^2^H‐IRMS performance and signal amplitude we used two samples: one was a transmethylated concentrated lipid extract of periphyton samples and the other a fish liver sample. Each FAME sample was measured four times on both GC columns. Peaks were selected and analyzed automatically as described above. The sample method resulted in column overload of the thinner 60 m column (0.25 mm ID, 0.25 μm FT) as indicated by broader and skewed peaks and a reduced H_2_ signal amplitude compared to the higher capacity 30 m column (0.32 mm ID, 1 μm FT) whereas the peak areas remained unaffected (Figure S1). Overall, more ^2^H peaks met the acceptable criteria for *δ* ^2^H determinations (>450 mV; >4 V s) using the 60 m GC column (15 versus 14); however, only two peaks were identified in the periphyton sample and the overall reproducibility for *δ* ^2^H was poorer than when using the 30 m column, which had an average standard deviation of 2.8‰ versus 2.0‰ (Table 1).

**TABLE 1:** Differences in analytical performance between standard and high‐capacity GC columns. A fish liver and one biofilm sample measured five times on both columns as described in the methods section. All baseline‐separated peaks >450 mV and >4 V s were used for analysis. Average amplitude and standard deviation of the *δ* ^2^H values were calculated for each peak. Mean standard deviation and mean amplitude represent the average of all eligible peaks

	Fish		Biofilm		Overall	
	30 m^a^	60 m^b^	30 m^a^	60 m^b^	30 m^a^	60 m^b^
Mean standard deviation *δ* ^2^H values (‰)	2.7	3.1	2.4	4.4	2.7	3.3
Max. standard deviation *δ* ^2^H values (‰)	5.0	6.1	6.1	5.2	6.2	6.1
Mean amplitude (V)	3.1	1.5	1.1	0.9	2.4	1.3
Max. amplitude (V)	9.0	3.8	2.0	0.9	9.0	3.8
Number of fatty acid peaks	9	13	5	2	14	15

^a^ 30 m/0.32 mm ID/1 μm FT.

^b^ 60 m/0.25 mm ID/0.25 μm FT.

One disadvantage of the standard thin 60 m column was a higher chance of peaks overlapping for some sample types due to the broad nature of FAME peaks. By contrast, H_2_ sample peak intensity increased by a factor of *ca* 2 by when using the 30 m HC‐GC column, thereby improving both the accuracy and precision of the *δ* ^2^H measurements. There was only a slight loss in chromatographic performance with the 30 m GC column, but for most of the FAME peaks of ecological interest a return of H_2_ signal to baseline levels between two analytes could be achieved.

### 3.2 Adjustment of environmental samples for *δ* ^2^H analysis

The abundance of fatty acid components in nature varies depending on organism, tissue or sample type, time, site, and other complex factors. We intended to develop a generalized robust method to conduct lipid *δ* ^2^H assays for a broad range of sample types, needed for studies in (aquatic) ecology, and to determine the required amount of sample for successful use in HC‐GC/^2^H‐IRMS analysis. For this process optimization we used a small subset of the archived samples (Figure S2).

### 3.3 Leaf litter

Leaf litter samples (*n* = 10) were collected during summer and autumn from streams or next to trees. On average, 2.5 ± 1.0 mg of total lipids could be extracted per litter sample when processing 35.5 ± 2.8 mg of freeze‐dried leaf material. In the GC and IRMS chromatograms,the most prominent peaks identified were C16:0 and ALA, with smaller amounts of C18:1 and LIN. The H_2_ signals for some peaks, especially C16:1, C18:0, and γ‐linoleic acid (GLA), were too low and hence two extracted leaf samples of the same origin had to be combined, which resulted in reproducible *δ* ^2^H values for C16 and C18 FAMEs. From these leaf litter results we recommend processing 70–100 mg of freeze‐dried leaves to obtain at least *ca* 5 mg of lipid extract.

### 3.4 Biofilm

Aquatic biofilm samples (*n* = 18) were obtained from submerged rocks by epilation and filtration. On average, 18.7 ± 6.8 mg of dry material yielded 0.4 ± 0.2 mg of lipids per sample. The most abundant fatty acids identified were C16:0 and C16:1. Trace amounts of EPA, identified in samples taken in stream headwaters, were insufficiently reliable for *δ* ^2^H quantification even after pooling of 4–5 similar biofilm samples, and hence require more material. On the other hand, EPA was more abundant in samples taken further downstream. C18:0, C18:1, LIN, GLA, ALA, and stearidonic acid (SDA) were detected in approximately equal amounts in all samples. The recommended sample mass for HC‐GC/^2^H‐IRMS of lipids in aquatic biofilms is 80–100 mg of dried material, to yield *ca* 2 mg of lipid extract per sample. If *δ* ^2^H values for other trace analytes such as EPA or DHA are required, then more material needs to be processed.

### 3.5 Aquatic invertebrates

Aquatic invertebrate samples (*n* = 19; including *Leuctra* sp., *Ecdyonurus* sp., and *Perla* sp.) contained similar lipid contents per gram of dry weight (128.6 ± 44.9 mg/g, average of all species). *Ecdyonurus* sp. were most lipid‐rich and resulted in 7.7 ± 2.8 mg of total lipid extract, followed by *Perla* sp. (4.0 ± 1.8 mg) and Leuctra (1.1 ± 0.5 mg). The GC and H_2_ signal intensities for most fatty acids were sufficient for *δ* ^2^H determinations in the case of *Ecdyonurus* sp. and *Perla* sp. (Figure 4), but required pooling of at least four individuals for *Leuctra* to achieve H_2_ amplitudes beyond 1000 mV for the FAMEs of interest.

### 3.6 Fish samples

Eight fish samples (two *Cottus gobio*, four *Oncorhynchus mykiss*, and two *Salmo trutta*) were analyzed. For each, the brain, liver, and muscle samples were subsampled. The average sample amount needed for lipid extraction was 6.4 ± 2.6, 13.2 ± 3.4, and 15.7 ± 3.1 mg for brain, liver, and muscle, respectively, resulting in a yield of 2.7 ± 1.5, 2.2 ± 0.7, and 0.8 ± 0.2 mg of total lipids. All samples provided sufficient GC and H_2_ signal intensities required for reliable *δ* ^2^H determination without any sample pooling. The brains had lower mass fractions of LIN, GLA, and ALA and quantification of SDA *δ* ^2^H values was not possible for two brain samples, one liver sample, and one muscle sample. For reliable quantification of fish tissue lipids, it is recommended to obtain at least 2 mg of total lipid extract per sample.

### 3.7 Pooled *δ* ^2^H values for leaves, biofilm, invertebrates, and fish

In leaves, the *δ* ^2^H isotope values of C18:0 (−142.2 ± 30.3 ‰), C18:1 (−131.0 ± 34.2 ‰), and LIN (−143.5 ± 11.7 ‰) differed significantly from C16:0 (−244.3 ± 15.2 ‰), C16:1 (−256.3 ± 109.4 ‰), and ALA (−209.2 ± 17.1 ‰). No reliable isotopic value for other fatty acids could be obtained for leaf litter.

In the biofilms, the *δ* ^2^H values of a greater number of fatty acid species could be determined. C16:0 (−300.9 ± 24.9 ‰) and C16:1 (−312.6 ± 25.7 ‰) were significantly depleted in deuterium compared to C18:0 (−235.6 ± 37.8 ‰), C18:1 (−216.0 ± 44.6 ‰), LIN (−165.7 ± 47.4 ‰), GLA (−220.8 ± 43.8 ‰), and ALA (−184.7 ± 48.0 ‰) though not compared to SDA (−276.0 ± 43.9 ‰) and EPA (−286.6 ± 43.0 ‰).

Pooled *δ* ^2^H data for the invertebrate samples revealed large H isotope differences between C16:0 and C16:1 (−271.2 ± 76.1‰ versus −329.3 ± 57.5‰), C18:0 and C18:1 (−212.9 ± 39.4‰ versus −268.3‰ ± 45.7‰), LIN and GLA (−181.4 ± 16.4‰ versus −238.6 ± 29.9‰), as well as ALA and SDA (−194.4 ± 43.1‰ versus −275.9 ± 22.2‰). ARA was the fatty acid most enriched in ^2^H (−108.8 ± 57.5‰), while *δ* ^2^H values of EPA (−268.6 ± 72.6‰) resembled those of C16:0.

Like invertebrates, pooled fish *δ* ^2^H data revealed large differences in H isotopic values between C16:0 and C16:1 (−322.8 ± 20.7 ‰ versus −381.7 ± 32.2 ‰), C18:0 and C18:1 (−239.2 ± 32.2 ‰ versus −321.2 ± 31.2 ‰), as well as ALA and SDA (−191.5 ± 33.75 ‰ versus −272.05 ± 40.3 ‰), but not between LIN and GLA (−222.6 ± 64.9 ‰ versus −242.9 ± 27.4 ‰). ARA (−89.2 ± 31.7 ‰) was the fatty acid most enriched in ^2^H, as with the invertebrates, while *δ* ^2^H values of EPA (−309.3 ± 27.5 ‰) and DHA (−310.3 ± 33.1 ‰) resembled those of C16:0.

### 3.8 Application of CSIA‐^2^H‐IRMS in ecological research: Examples of a river food web

Following optimization of compound‐specific deuterium analysis, more archived samples (for details, see Guo et al^35^) were measured with a focus on those collected in autumn 2016 at several reaches and tributaries of the River Ybbs catchment. In total, samples of 14 leaves, 35 biofilms, 156 aquatic insects (*Ephemeroptera* spp., *Tricoptera* spp., and *Plecoptera* spp.), 10 *Gammarus* spp., 3 *Plathelmintes* spp., and 127 fish (*Cottus gobio*, *Salmo trutta*, and *Oncorhynchus mykiss*) were used to explore the ecological potentials of ^2^H‐CSIA.

**FIGURE 3:**
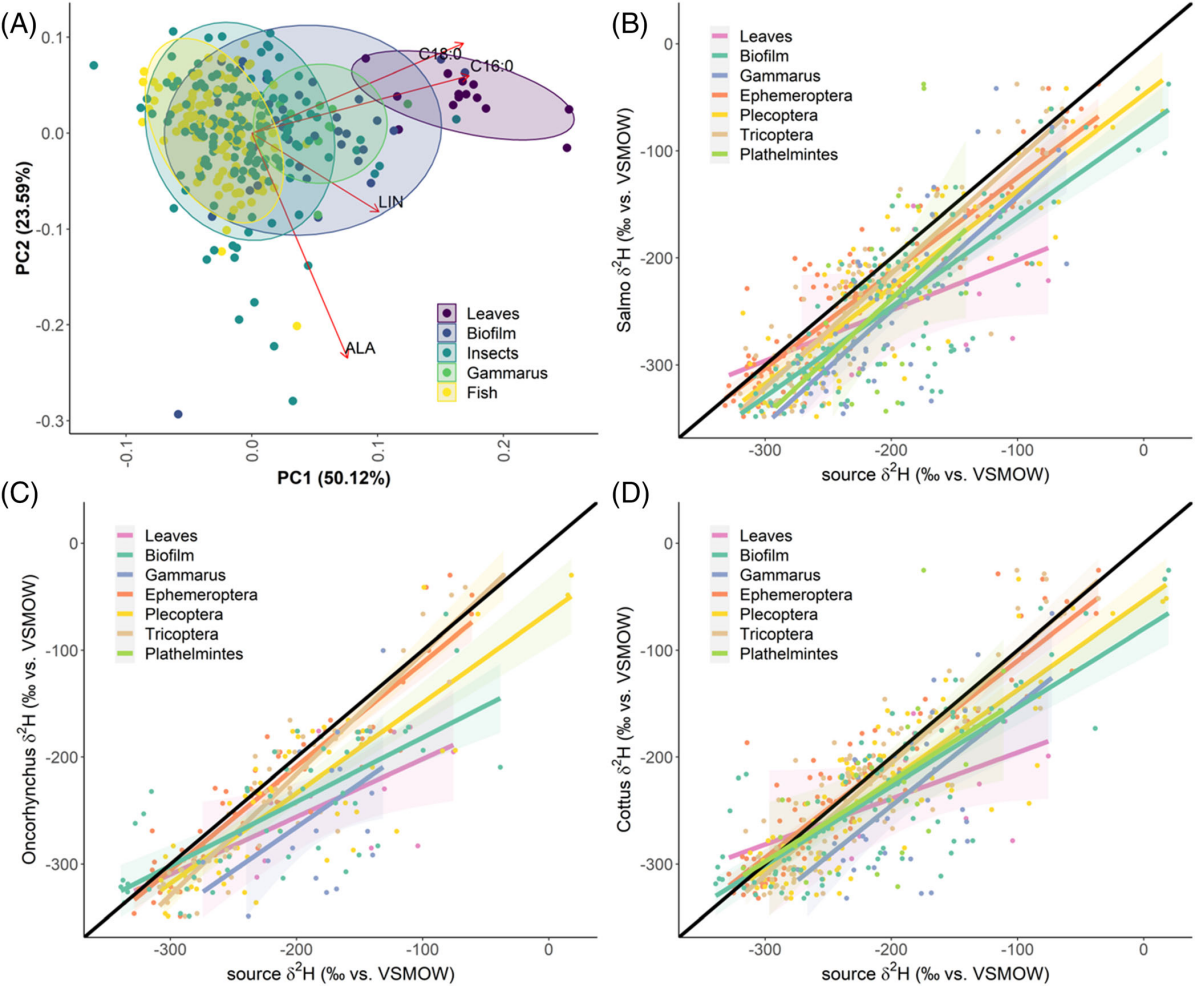
(A) PCA of *δ* ^2^H values of fatty acids abundant in all species (C16:0, C18:0, LIN, ALA) revealed a distinction between allochthonous and autochthonous dietary sources, with fish clearly resembling the H isotopic values of insects, but not of terrestrial food sources. H isotopic values of *Gammarus* spp. matched those of biofilms more closely than leaves. (B–D) Site‐specific CSIA correlation analysis provided further indication that aquatic insects are the main food source for fish in these streams, with *Gammarus* spp. potentially being part of the diet of *Cottus* spp. and *Salmo* spp. despite a negative offset of the intercept, which could be due to isotopic fractionation of fatty acids during digestion. In contrast, leaf litter could be clearly excluded as a fatty acid dietary source for fish. *δ* ^2^H values of all fatty acids identified in both source and consumer have been used for analysis

Deuterium values of fatty acids that were abundant in all samples (C16:0, C18:0, LIN, and ALA) resulted in different clustering of terrestrial and aquatic samples. PCA further revealed similar patterns for fish and insects, while *Gammarus* spp. resembled mainly biofilm, with a slight tendency towards the leaf cluster (Figure 3A). Comparison of the compound‐specific *δ* ^2^H values of the individual fish species with the corresponding *δ* ^2^H values of the potential diet, averaged for each sampling site, revealed a close to 1:1 correlation between fish and insects, mainly *Ephemeroptera* spp. and *Tricoptera* spp. Correlation of *δ* ^2^H values of *Plathelminthes* spp. and *Salmo trutta*, as well as *Salmo trutta* and *Cottus gobio* with *Gammarus* spp. also resulted in a regression slope close to 1; however, the intercept for correlation with *Gammarus* had a negative offset of −57 ‰ and −36 ‰, respectively (Figures 3B–3D; Table 2).

**TABLE 2:** Site‐specific correlation of potential dietary *δ* ^2^H values with those found in fish muscle

Fish	Diet	Intercept (‰)	Slope (‰)	*R* ^2^
*Cottus*	Leaves	−152.8	0.43	0.30
	Biofilm	−97.6	0.67	0.48
	*Gammarus* spp.	−57.5	0.94	0.34
	*Ephemeroptera* spp.	−35.9	0.86	0.67
	*Plecoptera* spp.	−73.6	0.76	0.56
	*Tricoptera* spp.	2.8	1.04	0.80
	*Plathelmintes* spp.	−142.9	0.41	0.15
*Oncorhynchus*	Leaves	−147.9	0.54	0.39
	Biofilm	−121.8	0.60	0.53
	*Gammarus* spp.	−102.2	0.82	0.24
	*Ephemeroptera* spp.	−14.7	0.97	0.87
	*Plecoptera* spp.	−64.7	0.84	0.69
	*Tricoptera* spp.	10.6	1.13	0.87
*Salmo*	Leaves	−155.0	0.47	0.27
	Biofilm	−78.1	0.84	0.66
	*Gammarus* spp.	−36.5	1.06	0.54
	*Ephemeroptera* spp.	−35.6	0.89	0.83
	*Plecoptera* spp.	−49.67	0.89	0.74
	*Tricoptera* spp.	−1.0	1.07	0.80
	*Plathelmintes* spp.	−18.1	1.10	0.20

**FIGURE 4:**
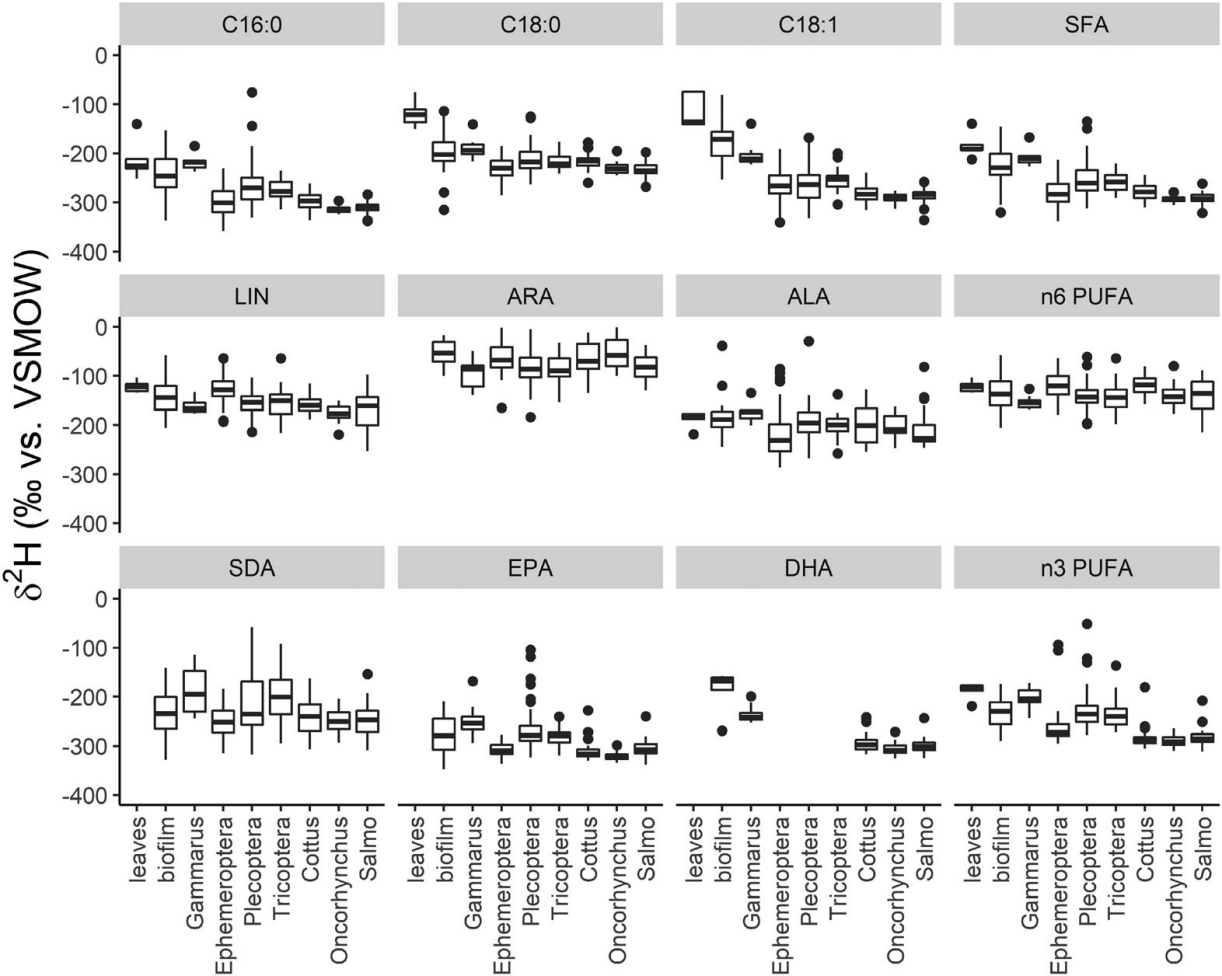
Pooled compound‐specific *δ* ^2^H values of terrestrial leaves, stream biofilms, invertebrates, and fishes from all sampling sites (also see Table S3). Saturated and mono‐unsaturated fatty acid (SFA), n‐6 PUFA and n‐3 PUFA values were calculated by weight‐averaging all identified corresponding fatty acids for an individual sample. Omega‐3 PUFA isotopic values of fish closest resemble those of *Ephemeroptera* spp., indicating that this group is an important diet source of essential fatty acids for fish

Further analysis of pooled data showed that *δ* ^2^H values of fish, especially for n‐3 PUFA, closely resembled those of *Ephemeroptera* spp. Leaf samples were clearly distinguished from other samples by their C18:0, C18:1, and LIN *δ* ^2^H values. Discrimination by ALA was less clear but could be achieved by using weight‐averaged (by peak area) values of all abundant n‐3 PUFA of an individual sample (Figure 4).

## 4 DISCUSSION

This study demonstrates that fatty acid‐specific *δ* ^2^H values provide more detailed information about dietary source retention in consumers of aquatic food webs than quantitative fatty acid analysis alone. This is because *δ* ^2^H values of the same fatty acids can differ among diet source, but consumers retain the *δ* ^2^H of dietary fatty acids selectively. Thus, using *δ* ^2^H of fatty acids of potential diet sources and consumers enables tracking of dietary fatty acid sources along food webs.

### 4.1 Sample preparation and analyses

Since the natural abundance of deuterium in organic materials is exceptionally low, it is recommended to optimize lipid sample preparations and GC conditions prior to isotope determinations to obtain sufficient lipid extract for characterization and to ensure sufficient H_2_ yield for IRMS. Sample size and collection requirements need to be considered in the study design in close cooperation with the laboratory to ensure enough material for reliable ^2^H‐CSIA is obtained. We investigated leaves, biofilms, invertebrates, and fish, collected from several sites for optimization of sampling conditions for future environmental studies, and we found that biofilms and small invertebrates pose the greatest challenge for obtaining enough lipid material. While the required amount of dried material used for lipid extraction will vary depending on the sample matrix, the amount of total lipids gained after lipid extraction should be at least 1 mg, and ideally 2–5 mg of extract per sample. For ^2^H‐CSIA, the sample should be pre‐concentrated under N_2_ or Ar flow to the optimal volume, at which good GC baseline separation and peak shape are still obtained, at least for the FAME peaks of interest, while their signal amplitude is maximized, ideally between 2 and 8 V. In our case, samples had to be pre‐concentrated to 30 μL, the minimal volume required for the autosampler, for optimal performance. The amount of lipid‐extracted sample injected onto a GC column is also limited by the column capacity, as column overload leads to peak broadening and skewed or overlapping peaks, which adversely affect the H isotope analyses. This can be circumvented by using a shorter (30 m) but high‐capacity GC column, i.e. with larger inner diameter and an increased film thickness. For quantification of fatty acid contents via the very sensitive GC‐FID method, good baseline separation for a large number of peaks (i.e. a good chromatographic resolution) is essential. Comparatively, the sensitivity of the hydrogen isotope ratio detector is rather low versus FID, accordingly reducing the number of ^2^H/H isotope‐resolvable FAME peaks in biological samples. Thus, in case of ^2^H‐CSIA of fatty acids, the benefit of an increased H_2_ signal intensity outweighs the minor loss in chromatographic resolution, and results in more reproducible *δ* ^2^H values for the compounds of interest. In this study, the lower number of identifiable FAME peaks in the fish tissue sample using the high‐capacity column during the performance evaluation was explained by a lower maximum GC temperature recommended by the manufacturer for this HC‐GC column (240°C versus 250°C), which led to peak broadening and loss of signal intensity for late‐eluting peaks such as DHA. This limitation can be overcome by using GC columns that endure higher temperatures without increased risk of column bleeding. For FAME <C22 the signal intensity was increased by a factor of about 2, while simultaneously preventing column overload. The higher signal‐to‐noise ratio in HC‐GC/IRMS improved the isotopic reproducibility and the number of peaks identified in complicated mixed samples compared to the standard 60 m column, which is a standard column typically used for lipid analysis. Finally, a further possibility to improve H_2_ signal intensity may be to use a lower temperature Cr‐based HTC reactor, which almost completely prevents the formation of alkane byproducts and provides quantitative, close‐to‐full‐conversion of organic compounds to hydrogen gas.^39^

### 4.2 Data processing modes influence analytical quality

An often‐neglected but very important topic in GC/IRMS is the determination and subtraction of background, which is a combination of instrumental noise and chemical (sample) noise. Typically, background levels rise if a strong temperature gradient is used. Ricci et al provided an excellent guide for IRMS data analysis of ^13^C, ^15^N, and ^18^O, and the principles of background calculation algorithms also apply to the more challenging GC/^2^H‐IRMS.^38^ Individual sample background calculation algorithms are routinely applied but are prone to errors by increased noise and variations in background, which is frequently amplified by sample concentration for HC‐GC/IRMS as seen in our ^2^H examples. It is therefore recommended to apply algorithms that accommodate background variations across the entire IRMS chromatogram, and by using dynamic background correction or the BaseFit algorithm. Application of these algorithms is more complicated since the calculation parameters such as the “step width” and “smoothing factor” need to be determined and adjusted for all samples within a run to ensure identical treatment. In addition, as demonstrated, a methanol correction is recommended to significantly reduce unexpected bias in the *δ* ^2^H values and provide comparability between analytical facilities.

### 4.3 Ecological implications, examples, and perspectives

Many studies using bulk tissue hydrogen isotope analysis demonstrated that *δ* ^2^H values typically show a much larger isotopic separation between dietary sources and consumers than ^13^C or ^15^N commonly used in trophic ecology.^30^, ^40^ Large differences in bulk tissue hydrogen isotopes in consumers are linked to the hydrogen isotope composition of environmental water controlled by precipitation patterns and evaporation processes, and to some extent is also translated to the compound‐specific level especially for nonessential molecules.^10^, ^16^ In terms of terrestrial and aquatic diets, large H isotopic differences of the order of approximately 100 ‰ are likely a result of: (i) algal H isotope fractionation during photosynthesis; (ii) H isotopic enrichment of water in terrestrial leaves due to transpiration particularly in ecosystems with low relative humidity and stomatal conductance; and/or (iii) because lipids are generally depleted in ^2^H, the higher lipid contents in algae compared to terrestrial leaves contribute more to overall bulk tissue deuterium depletion.^29^ As noted, the latter is caused by multiple steps during lipid synthesis,^33^ and thus, when using *δ* ^2^H, detailed knowledge of the physiological processes is required.^34^ Because alkyl H atoms do not exchange isotopes with ambient water, their *δ* ^2^H values remain unchanged after formation, except over timescales irrelevant for trophic ecology.^31^ These processes induce a great dynamic in H isotope fractionation in biological samples that manifests as different isotopic values at the fatty acid‐specific level, which can be exploited for a wide scientific range of ecological research.

We showed that *δ* ^2^H values of fatty acids (*δ* ^2^H_FA_) differed between autochthonous and allochthonous diet sources and can thus be used to track potential diets of consumers. The closest site‐specific *δ* ^2^H value correlation was between fish and insects, as expected, indicating the dietary importance of insects for fish. It turned out that *Ephemeroptera* spp. were the major diet source of fish as the *δ* ^2^H values of their n‐3 PUFA were more similar compared to *Plecoptera* spp. and *Tricoptera* spp. Insect *δ* ^2^H_FA_ were more closely related to *Oncorhynchus mykiss δ* ^2^H_FA_ than to *Salmo trutta δ* ^2^H_FA_, which were closer to unity for other benthic invertebrates, and could indicate different foraging strategies of these two salmonids (e.g. *Salmo trutta* feeding more on *Gammarus* spp.). In contrast, the correlation of fish and biofilm and especially leaf *δ* ^2^H values deviated significantly, indicating a negligible role for both as direct food source. Future studies might further explore the value of *δ* ^2^H_FA_ for food web analysis as a site‐independent marker.

When trying to identify potential diets, the isotopic values of *δ* ^13^C of essential fatty acids are reflected in consumers and it is likely that the same holds true for ^2^H.^41^, ^42^ Especially, both terrestrial plants and algae can synthesize LIN and ALA *de novo*; however, they are essential fatty acids for most consumers, which can only further convert them to long‐chain PUFA such as ARA, EPA, and DHA at a species‐dependent and usually limited rate.^43^, ^44^ Terrestrial plant‐ or algal‐derived n‐6 PUFA and n‐3 PUFA isotopic *δ* ^2^H values are thus unlikely to change significantly during trophic transfers, and consumers grazing entirely on algae should reflect similar FAME *δ* ^2^H values. However, little is yet known about how ^2^H is processed during lipid metabolism by consumers that may lead to alteration of the H isotopic compound‐specific value, as, for example, in benthivorous stream fish^35^ that derive DHA from precursors rather than directly from diet. The enzymatic processes required to convert precursors to DHA, often involving a series of elongation, desaturation, and even retro‐conversion (β‐oxidation) steps, are likely to involve changes in the ^2^H/H ratios of the affected fatty acids. Formation and dissolving of double bonds lead to exchange of H atoms in addition to H isotopic fractionation induced by the involved enzymes.^33^ Therefore, additional isotope research on consumer physiology for species of interest is needed, especially in combination with *δ* ^2^H and *δ* ^13^C‐CSIA, to significantly increase the isotopic resolution for food web analyses in the future.

## 5 CONCLUSIONS

This study provides a new reproducible protocol for the determination of *δ* ^2^H‐CSIA in biological lipid samples, and we show promising ecological applications such as resolving resource utilization in aquatic and terrestrial food webs (endogenous versus exogenous energy sources). As bulk *δ* ^2^H has been used for migration studies in the past, we also suggest *δ* ^2^H of fatty acids as a potential sensitive site‐specific marker. Finally, our method is applicable to a wide range of other ecological applications, such as fatty acid metabolism and routing in and within consumer tissues, food authenticity, and overall consumer response to diet change.

### PEER REVIEW

The peer review history for this article is available at https://publons.com/publon/10.1002/rcm.9135.

## Supporting information


**Figure S1.** **Direct comparison of** ^**2**^ **H‐GC‐IRMS using either a 30 m/0.32 mm I.D./1μm F.T. or a 60 m/0.25 mm I.D./0.25 μm F.T. column** (both VF‐WAXms, Agilent Technologies). The identical sample (concentrated transmethylated lipid extract of fish liver) was used. While shorter and thicker columns loose resolution, the higher capacity allows to load a much higher amount of sample onto the column leading to an increased signal by the IRMS, without skewed peaks due to column overload. While in both cases the area of the peaks is identical, the higher amplitude increases sensitivity and leads to more reproducible results.
**Figure S2. Examples of HC‐GC‐** ^**2**^ **H‐IRMS Chromatograms from different sample matrices.** Sample concentration was adjusted to obtain signal amplitudes of at least 1V for most peaks of interest. This required concentration and pooling of lipid extracts. C18:1isomers were reported as one peak, as no clear separation could be achieved.
**Table S3.** Mean plus standard deviation of compound specific δ^2^H values (‰ vs. VSMOW) of species from this study.

## Data Availability

Data can be accessed via: https://doi.org/10.5061/dryad.h70rxwdhj.
