# Supplementary material for: Compound‐specific stable hydrogen isotope (δ 2H) analyses of fatty acids: A new method and perspectives for trophic and movement ecology
Source: Rapid Commun Mass Spectrom. 2021 Jun 25;35(16):e9135. doi: 10.1002/rcm.9135 (PMC11478936; doi:10.1002/rcm.9135)
Supplement: Supplementary file 1 — Figure S1. Direct comparison of 2 H‐GC‐IRMS using either a 30 m/0.32 mm I.D./1μm F.T. or a 60 m/0.25 mm I.D./0.25 μm F.T. column (both VF‐WAXms, Agilent Technologies). The identical sample (concentrated transmethylated lipid extract of fish liver) was used. While shorter and thicker columns loose resolution, the higher capacity allows to load a much higher amount of sample onto the column leading to an increased signal by the IRMS, without skewed peaks due to column overload. While in both cases the area of the peaks is identical, the higher amplitude increases sensitivity and leads to more reproducible results. Figure S2. Examples of HC‐GC‐ 2 H‐IRMS Chromatograms from different sample matrices. Sample concentration was adjusted to obtain signal amplitudes of at least 1V for most peaks of interest. This required concentration and pooling of lipid extracts. C18:1isomers were reported as one peak, as no clear separation could be achieved. Table S3. Mean plus standard deviation of compound specific δ2H values (‰ vs. VSMOW) of species from this study. [file RCM-35-e9135-s001.docx]

**Compound-specific stable hydrogen (δ^2^H) isotope analyses of fatty acids: new perspectives for trophic ecology**

Supplementary Material

Matthias Pilecky^1,3^, Katharina Winter^1^, Leonard I. Wassenaar^2^ and Martin J. Kainz^1,3^

^1^WasserCluster Biologische Station Lunz, Inter-University Center for Aquatic Ecosystem Research, Dr. Carl-Kupelwieser Promenade 5, 3293 Lunz/See, Austria

^2^International Atomic Energy Agency, Vienna International Centre, Vienna, Austria 1400

^3^Department of BioMedical Research, Danube University Krems, 3500 Krems, Austria

E-mail:

MP: [matthias.pilecky@donau-uni.ac.at](mailto:matthias.pilecky@donau-uni.ac.at)

KW: [katharina.winter@wcl.ac.at](mailto:katharina.winter@wcl.ac.at)

MJK: [martin.kainz@donau-uni.ac.at](mailto:martin.kainz@donau-uni.ac.at)

LIW: l.wassenaar@iaea.org

Running Headline:

CSIA of Deuterium in trophic ecology

Keywords: deuterium, lipids, GC-IRMS, stable isotopes, food webs, ecology, mass spectrometry

# Figure S1


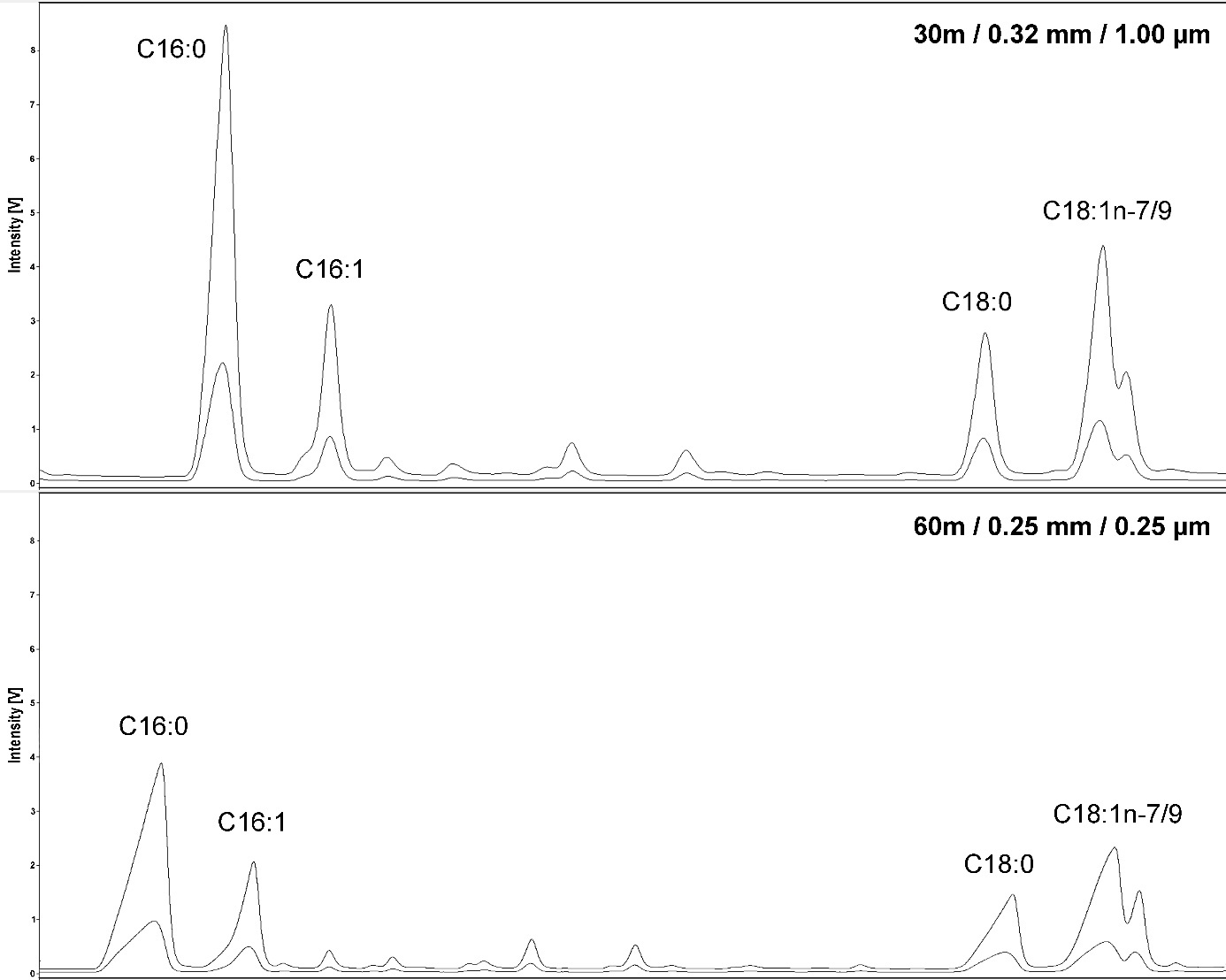


**Direct comparison of ^2^H-GC-IRMS using either a 30 m/0.32 mm I.D./1 µm F.T. or a 60 m/0.25 mm I.D./0.25 µm F.T. column** (both VF-WAXms, Agilent Technologies). The identical sample (concentrated transmethylated lipid extract of fish liver) was used. While shorter and thicker columns loose resolution, the higher capacity allows to load a much higher amount of sample onto the column leading to an increased signal by the IRMS, without skewed peaks due to column overload. While in both cases the area of the peaks is identical, the higher amplitude increases sensitivity and leads to more reproducible results.

# Figure S2


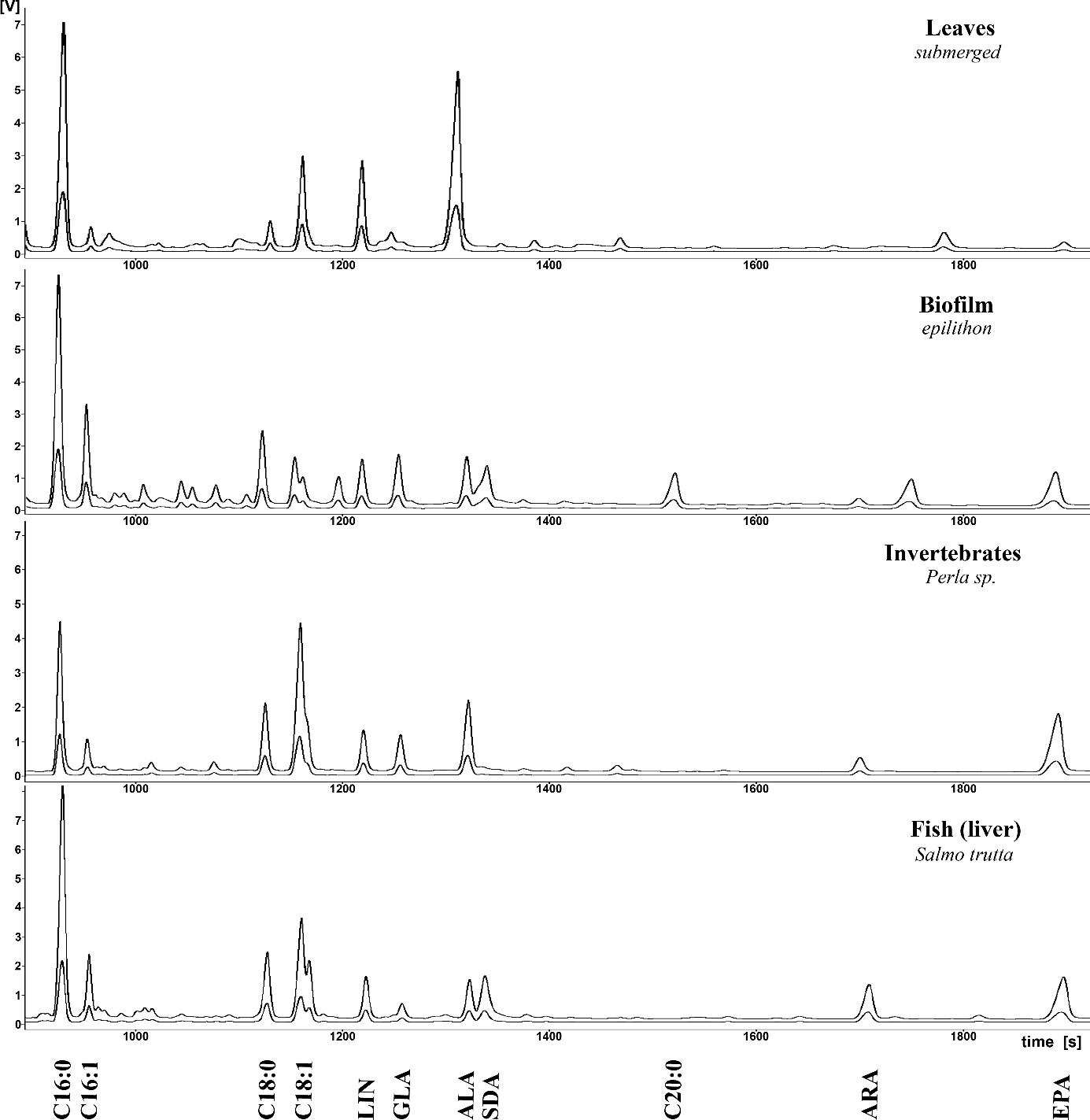


**Examples of HC-GC-^2^H-IRMS Chromatograms from different sample matrices.** Sample concentration was adjusted to obtain signal amplitudes of at least 1 V for most peaks of interest. This required concentration and pooling of lipid extracts. C18:1 isomers were reported as one peak, as no clear separation could be achieved.

# Table S3. Mean plus standard deviation of compound specific δ^2^H values (‰ vs. VSMOW) of species from this study.

|  | **leaves** | **biofilm** | ***Gammarus*** | ***Ephemeroptera*** | ***Plecoptera*** | ***Tricoptera*** | ***Cottus*** | ***Oncorhynchus*** | ***Salmo*** |
| --- | --- | --- | --- | --- | --- | --- | --- | --- | --- |
| C16:0 | -158.6 $\pm$ 48.6 | -245.6 $\pm$ 41.4 | -219.1 $\pm$ 15.1 | -298.2 $\pm$ 32.2 | -263.6 $\pm$ 46.1 | -275.2 $\pm$ 20.4 | -297.5 $\pm$ 16.8 | -314.0 $\pm$ 7.0 | -311.1 $\pm$ 11.0 |
| C18:0 | -119.3 $\pm$ 22.4 | -200.3 $\pm$ 37.1 | -189.9 $\pm$ 21.5 | -230.1 $\pm$ 19.9 | -211.3 $\pm$ 29.3 | -216.9 $\pm$ 16.8 | -216.6 $\pm$ 14.6 | -230.5 $\pm$ 11.7 | -233.1 $\pm$ 14.1 |
| C18:1 | -120.6 $\pm$ 41.5 | -178.8 $\pm$ 36.0 | -203.6 $\pm$ 24.4 | -263.3 $\pm$ 34.4 | -262.8 $\pm$ 38.7 | -254.5 $\pm$ 21.4 | -282.6 $\pm$ 15.8 | -291.1 $\pm$ 9.4 | -286.2 $\pm$ 13.8 |
| SFA | -148.3 $\pm$ 35.3 | -227.9 $\pm$ 36.9 | -209.0 $\pm$ 16.8 | -278.5 $\pm$ 31.4 | -252.2 $\pm$ 38.4 | -259.4 $\pm$ 18.7 | -279.0 $\pm$ 15.9 | -293.6 $\pm$ 6.5 | -292.1 $\pm$ 11.0 |
| LIN | -109.9 $\pm$ 27.6 | -143.7 $\pm$ 32.3 | -161.5 $\pm$ 14.9 | -128.8 $\pm$ 28.3 | -155.5 $\pm$ 29.0 | -153.9 $\pm$ 29.4 | -157.9 $\pm$ 17.0 | -176.8 $\pm$ 17.0 | -171.3 $\pm$ 36.7 |
| ALA | -173.4 $\pm$ 17.2 | -186.7 $\pm$ 36.0 | -175.5 $\pm$ 17.9 | -216.6 $\pm$ 51.3 | -192.3 $\pm$ 36.9 | -200.1 $\pm$ 21.1 | -200.6 $\pm$ 35.2 | -202.8 $\pm$ 21.3 | -213.0 $\pm$ 35.2 |
| n6 PUFA | -109.9 $\pm$ 27.6 | -137.3 $\pm$ 33.5 | -153.9 $\pm$ 13.1 | -119.2 $\pm$ 26.9 | -142.8 $\pm$ 29.3 | -143.1 $\pm$ 25.8 | -119.7 $\pm$ 19.7 | -142.0 $\pm$ 23.1 | -140.6 $\pm$ 34.5 |
| SDA | -240.2 $\pm$ 104.4 | -233.1 $\pm$ 51.1 | -187.1 $\pm$ 53.5 | -250.4 $\pm$ 35.4 | -205.1 $\pm$ 75.8 | -197.5 $\pm$ 50.5 | -241.0 $\pm$ 34.6 | -248.9 $\pm$ 23.2 | -248.1 $\pm$ 33.8 |
| n3 PUFA | -173.8 $\pm$ 17.3 | -233.8 $\pm$ 31.2 | -203.5 $\pm$ 24.4 | -262.8 $\pm$ 36.3 | -226.3 $\pm$ 42.0 | -236.3 $\pm$ 27.3 | -285.2 $\pm$ 18.0 | -289.3 $\pm$ 11.5 | -283.0 $\pm$ 17.3 |
| ARA | NA | -25.9 $\pm$ 54.5 | -97.5 $\pm$ 31.4 | -62.9 $\pm$ 34.4 | -61.8 $\pm$ 66.2 | -84.1 $\pm$ 37.2 | -64.4 $\pm$ 39.8 | -53.3 $\pm$ 32.5 | -81.8 $\pm$ 24.3 |
| EPA | NA | -277.7 $\pm$ 40.2 | -249.8 $\pm$ 36.3 | -307.1 $\pm$ 14.0 | -264.6 $\pm$ 47.0 | -281.9 $\pm$ 19.0 | -312.2 $\pm$ 16.1 | -320.4 $\pm$ 9.7 | -306.3 $\pm$ 17.1 |
| DHA | NA | -188.4 $\pm$ 46.4 | -235.6 $\pm$ 19.4 | NA | NA | NA | -295.0 $\pm$ 16.9 | -305.7 $\pm$ 12.2 | -299.6 $\pm$ 14.0 |
